# Estimation of Image Sensor Fill Factor Using a Single Arbitrary Image

**DOI:** 10.3390/s17030620

**Published:** 2017-03-18

**Authors:** Wei Wen, Siamak Khatibi

**Affiliations:** Department of Technology and Aesthetics, Blekinge Tekniska Högskola, 371 79 Karlskrona, Sweden; siamak.khatibi@bth.se

**Keywords:** fill factor, virtual image, image sensor, pipeline, virtual response function, sensor irradiance

## Abstract

Achieving a high fill factor is a bottleneck problem for capturing high-quality images. There are hardware and software solutions to overcome this problem. In the solutions, the fill factor is known. However, this is an industrial secrecy by most image sensor manufacturers due to its direct effect on the assessment of the sensor quality. In this paper, we propose a method to estimate the fill factor of a camera sensor from an arbitrary single image. The virtual response function of the imaging process and sensor irradiance are estimated from the generation of virtual images. Then the global intensity values of the virtual images are obtained, which are the result of fusing the virtual images into a single, high dynamic range radiance map. A non-linear function is inferred from the original and global intensity values of the virtual images. The fill factor is estimated by the conditional minimum of the inferred function. The method is verified using images of two datasets. The results show that our method estimates the fill factor correctly with significant stability and accuracy from one single arbitrary image according to the low standard deviation of the estimated fill factors from each of images and for each camera.

## 1. Introduction

Since the first developed digital camera equipped with charge-coupled device (CCD) image sensors in 1975 [1], the CCD digital camera has played a more and more important role in both normal life and scientific studies. This popularity is thanks to significant progress over the past decades in digital camera sensory techniques and image processing algorithms; especially achievements in increasing image resolution and improving low-light performance [2]. The progress of achievements are due to the reduction of the sensory element (the pixel size), improving the conversion of collected photons to electrons (the quantum efficiency), and using hardware techniques on the sensor [1,3]. However, the image quality is not affected only by the pixel size or quantum efficiency of a sensor [4]. As the sensor pixel size becomes smaller, this results in a smaller die size and a higher spatial resolution gain; all at the cost of a lower signal-to-noise ratio, lower dynamic range, and fewer tonal levels [5]. An effective method to improve the performance of a camera sensor and avoid the problems above is to increase the sensor fill factor, e.g., by arranging an array of microlenses on the sensor array [6,7]. However, due to the physical limitation in practical development and manufacturing of digital cameras, the fill factor of an image sensor cannot be 100% [2].

Since the increase of the fill factor by hardware solutions is difficult and limited, numerous image processing algorithms have been developed to solve the issue, e.g., by black level correction, noise reduction, white balance, tone reproduction, or histogram equalization for image enhancement [8]. The combination of such processes forms different image processing pipelines (IPPs) in a camera [9,10,11,12], and we discuss more of the IPPs in detail in Section 2. Today, almost all images we capture are processed by a type of pipeline in a camera. However, the quality of images is still far lower than what our visual system can perceive. At the same time, compared to human eyes that have a much larger range of luminance (10,000:1 vs. 100:1 cd/m^2^) [13], it is necessary for a camera sensor to have a larger fill factor with a die size to create more intensity levels for an image in the luminance range. Recently a new approach was presented [14] in which the fill factor is increased virtually, resulting in a significant extension of image tonal level and widening of the dynamic range. In the approach, the original fill factor is assumed to be known. However, this value is a confidential matter by most digital camera manufacturers. Even though, with the help of sensor pattern noise, the source of an image sensor can be identified [15], the information about the sensor fill factor remains unknown.

On the other hand, if we consider the capture process in an image acquisition pipeline, the charge collected by an image sensor element is proportional to the projected irradiance from its scene. The amount of irradiance Z on the image sensor element is varied by exposure time τ and fill factor ζ; i.e., Z≈L×τ×ζ where L is the radiance from the scene. Although the image sensor response to these optical variations is a linear function, most digital cameras apply a nonlinear mapping to the image sensor element before the captured image is written to the storage medium. The nonlinearity comes from the image acquisition pipeline (e.g., non-linear gaining, A/D conversion) and/or further processing in the IPP. In the problem of recovering the high dynamic range of photographs [16,17,18], the images are captured by the same camera (i.e., the same fill factor) and with different exposure times. Then, the response function of the nonlinear imaging process is recovered by a combination of the images into a composite radiance map.

In this paper, we propose a method to estimate the fill factor of a camera sensor from a captured image by the camera. In our method, from the captured image, a set of N new images with N number of fill factors are generated based on the proposed method in [14]. Then the virtual response function of the imaging process is estimated from the set of generated images which combines the images into a composite virtual radiance map. In this way, the exposure time τ is assumed to be constant, e.g., *K*, and irradiance Z becomes Z≈L×K×ζ; i.e., the virtual response function has certain relation to the variation of fill factors. Using this relation, and having a virtual response function, the actual fill factor is estimated. We verify our method and evaluate its stability and accuracy by using images of two cameras, Teli CS8620HCi (Toshiba Teli Corporation, Tokyo, Japan) and SONY XC-77 (SONY Corporation, Tokyo, Japan), which we could find the truth values of their fill factors [19,20]. The results show that our method can estimate the fill factor correctly with significant stability and accuracy from one single captured image. To the best of our knowledge, our approach is the first work that estimates the fill factor of an image sensor and proposes a method for estimation of a virtual camera response function.

The rest of paper is organized as follows; in Section 2 the image acquisition and processing pipeline in the camera are described; Section 3 explains the camera response function and then the methodology used for fill factor estimation is presented in Section 4 and Section 5; Section 6 explains the experimental setup, and the results are shown and discussed in Section 7; finally, we conclude and discuss potential future work in Section 8.

## 2. Image Acquisition and Processing Pipelines

**Figure 1:**
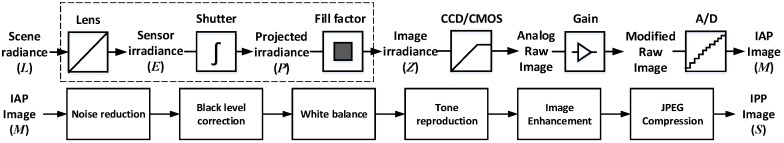
The image acquisition pipeline (**top**) and the image processing pipeline (**bottom**). The image acquisition pipeline shows the process of how scene radiance (L) becomes digital pixel values in an IAP image M, and the image processing pipeline shows the process of how an IAP image (M) can be processed further to obtain an IPP image (S).

The imaging process from the capturing of a scene to its display or storage in a medium generally undergoes two pipelines: the image acquisition pipeline (IAP) and the image processing pipeline, which are shown in Figure 1. As it is shown in the top of Figure 1, the scene radiance L (i.e., the energy flux emitted from the scene [21]) goes through the lens and is then projected on the image sensor with certain exposure time which is controlled by the shutter. The sensor irradiance E, in the figure, is the power per unit area of scene radiant energy L falling on the sensor. The power per unit area of the scene radiant energy L that is projected on the sensor is shown as the projected irradiance P in the figure. Additionally, due to the effect of the sensor fill factor, part of photons from the scene radiance is captured by the image sensor. The actual power per unit area of the scene radiant energy L that is captured and measured by the sensor is the image irradiance Z. The variation of the exposure time and the fill factor with respect to the input, sensor irradiance E, are linear processes. The acquisition of image irradiance from the scene radiance is shown in the top of Figure 1 by the dashed line box. Then photon-to-electron conversion is achieved by the image sensor, which is also a linear process with respect to each photon wavelength. It is noteworthy to mention that the quantum efficiency (the conversion of photons into electrons) is a nonlinear process with respect of different light wavelengths. We call the output of the image sensor an analog raw image, which is then amplified and normalized by a gain control; the modified analog raw image. The amplification can be nonlinear and the normalization is a nonlinear process. The modified analog raw image is sampled and quantized in an A/D conversion which results in the output image; as the IAP image M in Figure 1. The A/D conversion is a nonlinear process. As it was discussed above, there are both hardware and software solutions to enhance the sensor performance. Generally, the applied hardware solutions in the IAPs try to increase the number of captured photons; e.g., using a microlens array on the image sensor. On the other hand, the software solutions are mostly applied in the IPPs.

With performance of an image-processing pipeline, the produced digital IAP image is taken and a digital image is obtained that will then be viewed or undergo further processing before being saved to nonvolatile memory. This pipeline is a series of specialized algorithms in a certain order that adjusts image data in real-time and is often implemented as an integrated component of a system-on-chip (SoC) image processor. Without a well-designed processing flow, it is difficult to obtain satisfactory and stable image quality, even if suitable algorithms for individual stages are selected. Assigning these processing steps into appropriate stages in the IPP is a complicated task, and the ordering of IPP stages will dominate the final picture quality [9,10,11,12]. A simplified and typical IPP for a camera is shown at the bottom of Figure 1. The shown IPP is composed of the following parts: noise reduction, black level correction, white balance, tone reproduction, image enhancement, and image compression. Noise is reduced or eliminated due to its significant influence on dynamic range of the captured image [22]. Black level correction is applied for contrast correction by capturing an image of a completely black field [23]. Unrealistic white casts are removed by the white balance process; the process maintains color constancy of an image by removing color casts caused by an un-canonical illuminant [24]. The tone reproduction is used to produce realistic “renderings” of captured scenes by properly reproducing brightness and brightness differences [25]. The image enhancement is applied to modify attributes of an image and to make it more suitable for a given task and a specific observer [26], which mostly includes two processes: edge and contrast enhancement. The irrelevance and redundancy of the image data are reduced by the image compression to be able to store or transmit data in an efficient form, e.g., JPEG [27].

## 3. Camera Response Function vs. Virtual Camera Response Function

An IPP image, acquired by the image acquisition and processing pipelines, shown in Figure 1, is related to scene radiance by a nonlinear mapping function. The knowledge of the scene radiance reveals physical properties of the scene caused by the interaction of light with scene objects. The amount of scene radiance is varied as the amount of light or scene objects are varied. Thus, the nonlinear mapping function which relates an IPP image to scene radiance is a partial camera response to the limited range of the scene radiance. Generally, to obtain the camera response function we use an image of a uniformly illuminated chart with patches of known reflectance, such as the Macbeth chart, as it is done in [28]. Nevertheless, placing a chart in the scene is quite inconvenient or difficult in practical applications. Thus, obtaining the camera response function is a difficult task. On one hand, a single captured image, the IPP image S, is related to a partial camera response function and, on the other hand, it is almost impossible to rearrange a scene, e.g., with a Macbeth chart, to include all scene radiance variations. Let us now, in more detail, consider what happens during the formation of an IPP image. The sensor irradiance, *E*, is acquired in relation to scene radiance *L* as [29]:

(1) $$E_{i}=E\left(i,\lambda \right)=\frac{\pi }{4}{\left(\frac{d}{h}\right)}^{2}T\left(\lambda \right){\left(\cos\varphi \right)}^{4}L\left(\frac{i}{m},\lambda \right)$$

where *h* is the focal length of the imaging lens, *d* is the diameter of aperture, φ is the angle subtended, m is the lens magnification, T(λ) is the transmissivity of the lens, i is the spatial index of each sensor element, and λ is the light wavelength. Accordingly, the image irradiance *Z* is $Z_{i}=E_{i}\tau \zeta$:

The optimized method to preserve the existing dynamic range of the scene radiance *L* in the acquired image *Z* is to choose the correct exposure time for the radiation of each scene point and having a 100% fill factor. As the exposure time for capturing an image is fixed for all scene points and the fill factor of a camera is generally far from 100% [14], we should surely assume that only part of the dynamic range of *L* is captured. The nonlinear function of f, which is caused by components in IAP and IPP, maps the image irradiance *Z* to the output of IAP; the IAP image M, as:

(2) $$M_{i}=f\left(Z_{i}\right)=f\left(E_{i}\tau \zeta \right)$$

by assuming that the sensor output increases monotonically, or at least semi-monotonically, with respect to $Z_{i}$, the function f is invertible. Then the partial camera response function $g_{par}$ is:

(3) $$g_{par}=f^{-1}\left(M_{i}\right)=E_{i}\tau \zeta .$$

The knowledge of used nonlinear components and their response functions for each camera is generally difficult to acquire, which forces us to search for other solutions. One solution is to vary the exposure time and capture a set of images with the same camera; i.e., the fill factors are the same. Accordingly, Equation (2) becomes:

(4) $$g\left(M_{il}\right)=f^{-1}\left(M_{il}\right)=\zeta E_{i}\tau_{l},$$

where g is the camera response function and l is the exposure time index. We should note that we called g as the camera response function due to the assumption of having enough variations of exposure times; i.e., the $g_{par}$ goes to g by a greater number of captured images with different exposure times. This solution is used in the recovery of high dynamic ranges of images [30,31]. Another solution to the problem expressed in Equation (2) is to use a single captured image and vary the fill factors to obtain a set of images; i.e., the exposure time remains the same and the images are generated virtually [14]. Accordingly, this time Equation (2) becomes:

(5) $$g_{v}\left(Mv_{ij}\right)=f^{-1}\left(Mv_{ij}\right)=\tau E_{i}\zeta_{j},$$

where $g_{v}$ is the virtual camera response function, j is the fill factor index, and Mv is the generated virtual image. We noted the camera response function as the virtual one due to our proposed method of using virtual images. For the recovery of $g_{v}$ from Equation (4) we used the same methodology in [32] in which a similar problem is discussed in relation to exposure time variation. Accordingly, by taking the natural logarithm of both sides of Equation (5), and using the set of equations arising from it, the best recovery (in sense of least square error) of $g_{v}$ and $E_{i}$ is computed from:

(6) $$\theta =\overset{M}{\underset{i=1}{\sum }}\overset{N}{\underset{j=1}{\sum }}{\left[w\left(Mv_{ij}\right)\left\{g_{v}\left(Mv_{ij}\right)-lnE_{i}-ln\zeta_{j}-ln\tau \;\right\}\right]}^{2}+\lambda \overset{Mv_{max}-1}{\underset{Mv=Mv_{min}+1}{\sum }}{\left[w\left(Mv\right)g^{\prime \prime }\left(Mv\right)\right]}^{2},$$

where −lnτ as constant value is ignored, M is the number of pixel locations, N is the number of virtual images, λ is an optional scaler and is chosen in relation to expected noise in $Mv_{ij}$, and $g^{\prime \prime }$ is used as $g^{\prime \prime }\left(Mv\right)=g\left(Mv-1\right)-2g\left(Mv\right)+g\left(Mv+1\right)$. w(Mv) is a weighting function:

$$w\left(Mv\right)=\{\begin{array}{ll} Mv-Mv_{min} & Mv\leq Mv_{mid}\\ Mv_{max}-Mv & Mv>Mv_{mid} \end{array}\;,$$

To emphasize the smoothness and fitting terms towards the middle of the response curve, according to Equation (6), we find $g_{v}$ and $E_{i}$ using pixel values from all available generated images; a mapping which constructs the sensor irradiance up to a factor of scale as:

(7) $$g_{v}\left(Mv_{ij}\right)=\frac{\sum_{j=1}^{N}w\left(Mv_{ij}\right)\left(lnE_{i}-ln\zeta_{j}\right)}{\sum_{j=1}^{N}w\left(Mv_{ij}\right)},$$

## 4. Relation of Fill Factor to the Virtual Camera Respond Function

In the previous section, we showed that by assuming a known fill factor $\zeta_{j}$ a virtual image $Mv_{ij}$ is generated and the sensor irradiance is mapped to the virtual image by a corresponding virtual camera response function (see Equation (7)). Having a set of N virtual images from N assuming known fill factors, a more enhanced virtual camera response function is obtained (depending on the number N) as:

(8) $$g_{v}\left(Mv_{ij}\right)=\overset{N}{\underset{j=1}{⋃}}g_{v}\left(Mv_{ij}\right),$$

The above equation expresses that each corresponding virtual camera response function $g_{v}\left(Mv_{ij}\right)$ to a fill factor $\zeta_{j}$ recovers only partially the relation between the sensor irradiance and all generated virtual images. The final estimated virtual camera response function will be the union of each virtual camera response function corresponding to its fill factor. At the same time, we can consider that between each $g_{v}\left(Mv_{ij}\right)$, by using Equation (5), it yields:

(9) $$g_{v}\left(Mv_{ik}\right)=\frac{\zeta_{k}}{\zeta_{j}}g_{v}\left(Mv_{ij}\right),$$

The pixel intensity values of each Mv is proportional to the fill factor. Thus, for their virtual camera response function it yields:

(10) $$g_{v}\left(Mv_{ij}\right)=\zeta_{j}I_{ij},$$

where $I_{ij}$ is the pixel intensity value at an index position of i with a fill factor index of j.

## 5. Fill Factor Estimation

**Figure 2:**
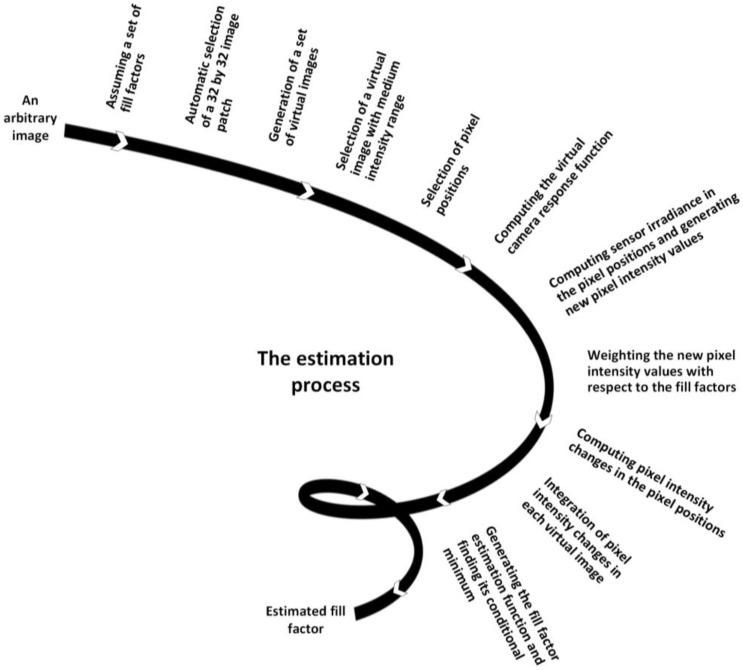
Flowchart of the fill factor estimation.

In this section, we explain how, from a single arbitrary captured image by an unknown camera (i.e., the fill factor is unknown), the fill factor is estimated by steps which are shown in Figure 2.

Previous experience in estimation of camera response by exposure time variation [32] shows that a group of pixels with different intensities are necessary to be selected in IPPs images; i.e., having more variations in pixel intensities, the estimation becomes more accurate. In our work a 32 × 32 patch of pixels with rich variation of pixel intensities is selected automatically; i.e., the range of pixel intensity variations of all possible 32 × 32 patches are examined by calculating the number of tonal levels in their histograms to select the patch. Then the patch is normalized by removing the offset of its histogram from zero, and a set of N virtual images is generated by the proposed method in [14], assuming knowing a fill factor from a set of fill factors between 22% and 81%. According to the method, the process of virtual image generation is divided into three steps of (a) projecting the pixel intensity onto a new grid of subpixels; (b) estimating the values of subpixels based on a local learning model; and (c) estimating the new pixel intensity by decision-making. The three steps are elaborated below:

(a) A grid of virtual image sensor pixels is designed. Each pixel is divided into 30 × 30 subpixels. According to the hypothetical fill factor ζ, the size of the active area is *A* by *A*, where $A=30\times \sqrt{\zeta }$. The intensity value of every pixel in the original image is assigned to the virtual active area in the new grid. The intensities of subpixels in the non-sensitive areas are assigned to be zero.
(b) The second step is to estimate the values of the subpixels in the new grid of subpixels. Considering the statistical fluctuation of the incoming photons and their conversion to electrons on the sensor, a statistical model is necessary for estimating the original signal. Bayesian inference is used for estimating every subpixel intensity which is considered to be in the new position of resampling. Therefore, the more subpixels that are used to represent one pixel, the more accurate the resampling is. By introducing the Gaussian noise into a matrix of selected pixels, and estimating the intensity values of the subpixels at the non-sensitive area with different sizes of active area by local modeling, a vector of intensity values for each subpixel is created. Then each subpixel intensity is estimated by the maximum likelihood.
(c) In the third step, the subpixels are projected back to the original grid. To obtain the intensity value of the pixels in the original grid, the intensity value of a pixel in the new grid is the intensity value which has the strongest contribution in the histogram of belonging subpixels. The corresponding intensity is divided by the fill factor for removing the fill factor effect to obtain the pixel intensity.

The generated virtual images are normalized, using their histograms, as follows:

$$I_{output}=\frac{\left(I_{input}-\min\left(I_{input}\right)\right)}{max\left(I_{input}\right)-\min\left(I_{input}\right)}\times Rn$$

where $I_{input}$ and $I_{output}$ represent the input and output images, respectively, and Rn is the intensity range of the input image, which is defined as:

$$Rn=\max\left(I_{input}\right)\times \zeta ,$$

Thus, the tonal range of each virtual image is proportional to its fill factor. By this way the N virtual images have N ranges of intensities. The virtual image with a medium range of intensities is chosen for obtaining a set of pixels in which each pixel has different intensity values. In the case of having several pixels with the same intensity, one of them is selected randomly. The position of each pixel in the set (i.e., the spatial index of each sensor element) is used for all of the virtual images (i.e., $Mv_{ij}$). According to the solution of Equations (6) and (8), the virtual camera response function, $g_{v}\left(Mv_{i}\right)$, using $Mv_{ij}$ is obtained. Actually, this function is an optimized solution having $Mv_{ij}$ (see Section 3) and, at the same time, it is a result of all partial virtual camera responses (see Equation (8)). This means that if each partial virtual response function shows the relation of $E_{i}$ to each $Mv_{ij}$ (such as showing part of a larger picture), the virtual camera response function shows the relation of $E_{i}$ to all $Mv_{ij}$, which generates new pixel intensity values for each of $Mv_{ij}$. Let us elaborate this by an example. Assume for a pixel position i in ten virtual images we have ten different intensity values. By having $g_{v}\left(Mv_{i}\right)$ and $E_{i}$, we obtain ten new intensity values which should increase proportionally to $\zeta_{j}$; this follows what we obtained in Equation (9). By compensating $1/\zeta_{j}$ we should expect all new pixel intensity values for each of $Mv_{ij}$ to have the same amount of changes. However, the single arbitrary image has an actual fill factor which is unknown and, therefore, we suggest using a set of assumed fill factors. If any assumed fill factor is less than the actual fill factor, we obtain the new pixel values, which are overestimated. If any fill factor is assumed more than the actual fill factor, we obtain underestimated values for the new pixel intensities. This means the changes in the estimation of the new pixels follow a non-linear function. We use this nonlinearity to estimate the actual fill factor. The non-linear function is obtained as follows: (a) the $E_{i}$ of i pixel position for each $Mv_{ij}$ is estimated and weighted by $1/\zeta_{j}$; (b) for each $Mv_{ij}$, and for each pixel position, the difference between the pixel intensity and the estimated $f\left(E_{i}\right)$ is calculated where function f is the inverse of $g_{v}$; (c) all N distances are normalized with respect to the minimum distance min(d), where $d=\sum_{j=1}^{N}Mv_{ij}-f\left(E_{i}\right)$; (d) the sum of all distances related to the pixel positions for each virtual image (related to each fill factor) is calculated; (e) the function of fill factor estimation (FFE), Y, is generated which relates the set of assumed fill factors to the sum of all the distances as:

(11) $$Y_{j}=\overset{M}{\underset{i=1}{\sum }}\frac{Mv_{ij}-f\left(E_{i}\right)}{min\left(d\right)}=\overset{M}{\underset{i=1}{\sum }}\frac{\zeta_{j}I_{ij}}{\zeta_{r}},$$

(f) the conditional minimum of the FFE is computed using the maximum local standard deviation, which results in the fill factor estimation.

In summary, a single arbitrary image is used to generate a set of virtual images. The virtual camera response function is obtained to estimate the pixel intensity values of the virtual images, accordingly. The rate of pixel intensity changes is used to estimate the actual fill factor.

## 6. Experimental Setup

**Figure 3:**
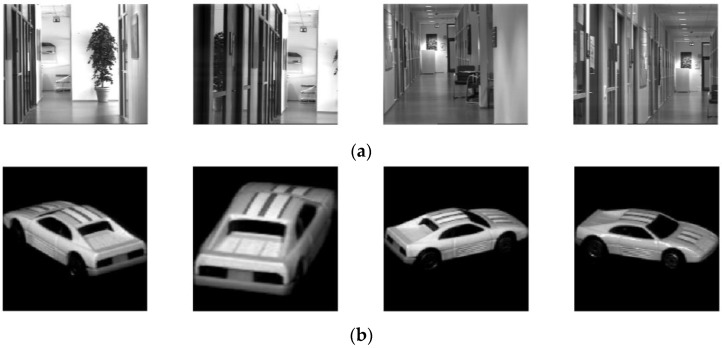
Image examples from two databases. (**a**) shows images taken by a Teli CS8620HCi and (**b**) shows images taken by a SONY XC-77.

Two groups of images from two cameras (Teli CS8620HCi and SONY XC-77) are used for the sensor fill factor estimation. We recall these datasets as the Teli dataset and SONY dataset. The ground truths of the camera fill factors are found with extensive research. The Teli dataset images are captured from the indoor and outdoor scenes at different positions to ensure the images have different histograms or, in another word, have different scene radiance maps, and the frame grabber of the Meteor-II/Multi-channel from Matrox is used to digitize the analog captured images. The sensor exposure time is selected by the camera automatically, and the gains in the camera and frame grabber are set at AGC (automatic gain control). With such a camera setup, twelve images are captured, of which four of them are shown in the first row of Figure 3. Then, the same procedure for fill factor estimation in Section 3 is applied ten times for each of the images. According to its known fill factor, which is around 39% [19], ten fill factors are assumed in the set of fill factors, which are 28%, 32%, 36%, 40% 44%, 49%, 54%, 59%, 64%, and 69% for generating the virtual images.

The images of the SONY dataset are from the COIL-20 image databases, as proposed in [33]. The database is used for evaluating the repeatability and the accuracy of our method due to its extensive number of images. In this database, there are 1440 grayscale images of 20 objects captured by a SONY XC-77 CCD camera. The objects have a wide variety of complex geometric and reflectance characteristics so that each of the images has a unique histogram. Eighty images of five objects are randomly selected from COIL-20 database and used to form the SONY dataset. Four images of one object from this database are shown in the second row of Figure 3. According to [20], the fill factor of the sensor in the SONY XC-77 is close to 70%. Thus, the set of fill factors that compare to the previous set is extended to 81% by having another two fill factors of 75% and 81%.

## 7. Results and Discussion

**Figure 4:**
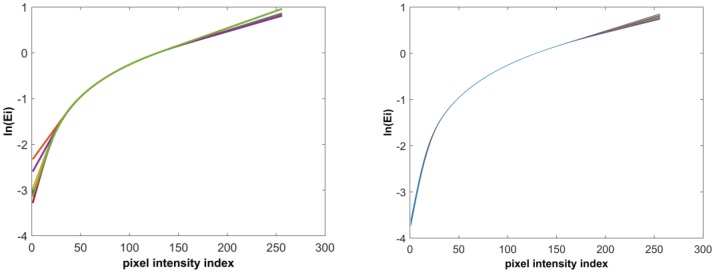
Estimated virtual camera response functions from generated virtual images. **Left**: Teli dataset; **Right**: SONY dataset.

The virtual camera response functions recovered from images of the two datasets are shown in Figure 4, where the left and right show the functions for the Teli dataset and the SONY dataset, respectively. The virtual camera response functions show the nonlinear process between intensity values of virtual images and the variation of fill factors which reflects the camera process on the captured image. The recovered response functions from images of the Teli dataset, shown on the left of Figure 4, show more variations where the intensity values are low. We believe this is due to the sensitivity of the virtual image generation process, in which having a lower known fill factor results in a variation of lower intensity values in the generated virtual image, is greater than having a higher fill factor. Compared to the camera response function recovered by the method in [32], the function estimated in our experiment avoids the saturation part where the intensity values are high. This makes the function purely monotonic, which is beneficial to the extension of the dynamic range and the increase of the tonal levels.

**Figure 5:**
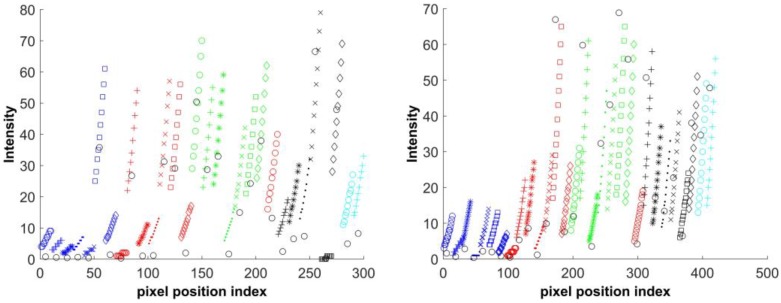
Two example results of the sensor irradiance $E_{i}$ calculations according to Equation (7). **Left**: Teli dataset; **right**: SONY dataset. The black circles represent the estimated sensor irradiance $E_{i}$ for the respective pixel position index of i . Each set of ten or twelve intensity values for each pixel position; obtained from fill factor variation, are shown with certain color and shape respectively from Teli or SONY dataset.

**Figure 6:**
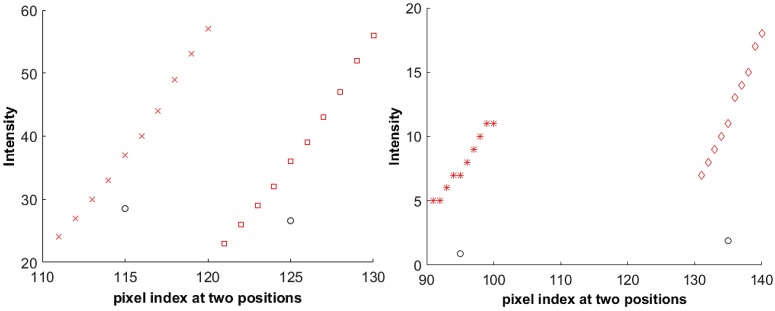
The examples of inliers (**left**) and outliers (**right**) of image irradiance points. The black circles represent the estimated sensor irradiance $E_{i}$ for the respective pixel position index of i . Each set of ten intensity values for each pixel position; obtained from fill factor variation, are shown with certain color and shape.

**Figure 7:**
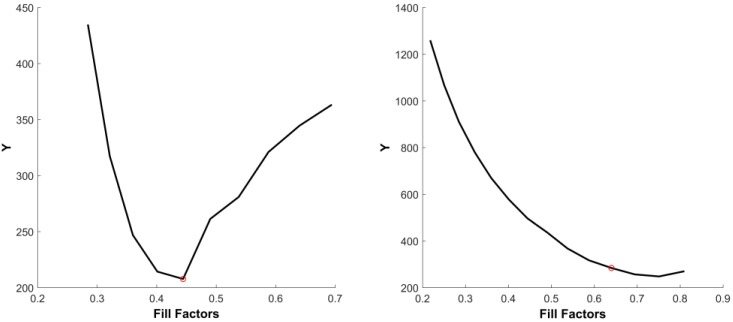
Two example results of fill factor estimation function, Y. **Left**: Teli dataset; **right**: SONY dataset. The red stars show the estimated fill factors.

The sensor irradiance $E_{i}$ is calculated by the inverse of the recovered virtual camera response from Equation (7). Two example results of such a calculation are shown in Figure 5 for two datasets. All of the marks in the figure, except the black circles, represent the intensities of each pixel position on the virtual images. There are ten or twelve intensities for each pixel position; i.e., with respect to the set of fill factors. Figure 5 shows the intensity changes at thirty pixel positions for the Teli dataset and SONY dataset on the left and right of the figure, respectively. The black circles represent the values of the sensor irradiance $E_{i},$ estimated from the virtual camera response function. The points marked with the same color and symbol form a space. Accordingly, each black circle corresponds only to one of the spaces. The black circles that are far away from their corresponding space are considered as the outliers. Figure 6 shows example of inliers and outliers of the presented data in Figure 5 on the left and right, respectively. In the left of Figure 6, the $E_{115}$ and $E_{125}$ are close to the pixel intensities of the virtual images. They are considered as inliers and are used for further estimation of the actual fill factors. On the contrary, the black circles, representing $E_{95}$ and $E_{135}$, are considered as outliers in the right of Figure 6 due to being far from the space of the pixel intensities. The pixel intensity that has the smallest distance to the corresponding $E_{i}$ in each space is considered as the reference; i.e., its corresponding fill factor is used as the reference for later fill factor estimation, as was discussed in Section 3. According to Equation (11), each of the $E_{i}$ that are marked as the inlier in its corresponding space is used to obtain the $Y_{j}$, the sum of the differences between the pixel intensity and the estimated $f\left(E_{i}\right)$, as it was explained in Equation (11). The functions of fill factor estimation, Y, for the presented data in Figure 5 are shown in Figure 7. The conditional minimum of each function of the FFE is shown with red in the figure for Teli and SONY cameras on the left and right, respectively.

According to Section 4, the new pixel intensity values of each image are proportional to the fill factor. Thus, the ratio of intensities at the same pixel position is the same as the ratio of the fill factors of the corresponding pixel intensities. By applying the inverse of the fill factor as the weighting factor in the computation of the FFE, according to Section 4, we should expect a constant function of the FFE. However, as the two examples of both of the FFEs in Figure 7 show the same pattern, that when the assumed fill factor is far away from the actual one, the value of Y is over- or under-estimated. When it is close to the actual sensor fill factor, the FFE becomes flat. For the estimation of the fill factor, we used this pattern by computing a conditional minimum of the FFE where the maximum of the local standard deviation of the FFE is the condition.

**Figure 8:**
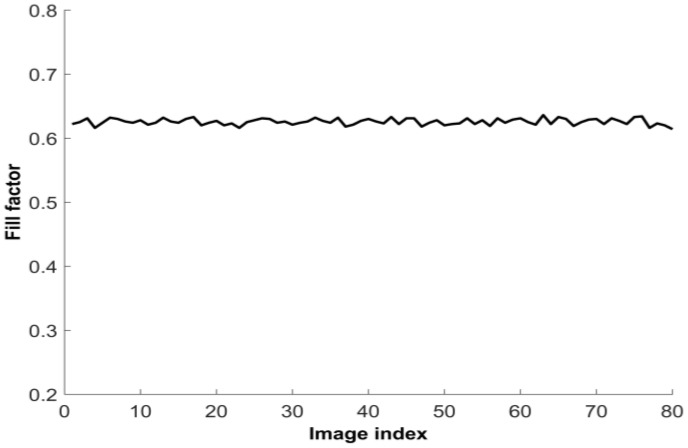
The results of the fill factor estimation for the SONY dataset.

The results of estimated fill factors for Teli dataset (from ten images) are shown in Table 1 and Table 2, showing part of the results for the SONY dataset (only from ten images). The results in the two tables show that, for each camera, the estimated fill factor varies between two fill factors’ values, which are 40%, 44%, and 59%, 64% for Teli and SONY cameras, respectively. For the Teli camera, the results of 29% of the tests show an estimated fill factor of 40%, and 71% of which the estimated fill factor is 44%. Then the following equation is used for calculating the final estimation of the fill factor:

(12) $$FF=\overset{N}{\underset{n=1}{\sum }}f\left(n\right)\;\times \;P_{r}\left(n\right),$$

where FF is the final estimated fill factor, f(n) and n represent the fill factor and its sequence number which varies from one to ten, $P_{r}\left(n\right)$ represents the probability of each fill factor, and *N* is the number of used images from a dataset. The results shown in Table 1 and Table 2, for each of the images, are obtained by applying the estimation process on each image 30 times, pursuing more solid statistical results. It should be noted that a generated virtual image varies in each time of the generation. Then the estimated fill factor from each image, which is shown in the row labeled as ‘Final’ in Table 1 and Table 2, is calculated using Equation (12). The final estimated fill factors from every image in the SONY dataset are plotted in Figure 8. According to the estimation from all of the images of each camera dataset, the fill factor of the Teli camera and SONY XC-77 are estimated as 43% and 63%, respectively. These fill factors were estimated as close to 39% and 70% for the Teli [19] and SONY camera [20], respectively. The standard deviations of the estimated fill factor from the whole image dataset of the Teli dataset and SONY dataset are 0.4% and 0.5%, respectively, which indicate that the results of the estimation are highly stable. The response functions estimated from the images of the SONY dataset show a larger range of irradiance, shown in Figure 4, indicating that the camera sensor from the SONY-XC77 is more sensitive than the Teli CS8620HCi, due to it having a larger fill factor.

## 8. Conclusions

In this paper, we propose a method to estimate the fill factor of a camera sensor from an arbitrary single image. In our method, the virtual response function of the imaging process is recovered from a set of images which are generated by a set of assumed fill factors using the proposed method in [14]. Then, by using the inverse of the virtual response function and the sensor irradiance, the global intensity values of the virtual images are obtained, which are the results of fusing the virtual images into a single, high dynamic range radiance map. A non-linear function is inferred from the original and global intensity values of the virtual images. The fill factor is estimated by the conditional minimum of the inferred function. The method is verified by using images of two datasets, captured by Teli CS8620HCi and SONY XC-77 cameras. The results show that our method is able to estimate the fill factor correctly with significant stability and accuracy from one single arbitrary image. The low standard deviation of the estimated fill factors from each image, and for each camera, confirms the stability of our method. At the same time, the experimental results also prove the feasibility of the estimation of the camera response function (a virtual one) with a single image, which can be useful for extending dynamic range of an image in the future. The images used in this work are all monochrome and captured by CCD cameras, and the investigation of the current problem using color images and CMOS cameras will be very interesting in the future.

## Figures and Tables

**Table 1:** Part of the results of fill factor estimation for the Teli dataset.

Images	No. 1	No. 2	No. 3	No. 4	No. 5	No. 6	No. 7	No. 8	No. 9	No. 10	Total	Percent
**FF 40%**	5	8	10	9	12	11	7	8	15	4	88	29%
**FF 44%**	25	22	20	21	18	19	23	22	15	26	212	71%
**Final**	43%	43%	43%	43%	42%	42%	43%	43%	42%	43%	43%	

**Table 2:** Part of the results of fill factor estimation for the SONY dataset.

Images	No. 1	No. 2	No. 3	No. 4	No. 5	No. 6	No. 7	No. 8	No. 9	No. 10	Total	Percent
**FF 59%**	13	11	8	15	10	5	6	18	11	13	110	37%
**FF 64%**	17	19	22	15	20	25	24	12	19	17	190	63%
**Final**	62%	62%	63%	62%	62%	63%	63%	61%	62%	62%	62%	
